# The evolution of mating type switching

**DOI:** 10.1111/evo.12959

**Published:** 2016-06-17

**Authors:** Zena Hadjivasiliou, Andrew Pomiankowski, Bram Kuijper

**Affiliations:** ^1^ CoMPLEX, Centre for Mathematics and Physics in the Life sciences and Experimental biologyUniversity College LondonGower StreetLondonUnited Kingdom; ^2^ Department of GeneticsEvolution and Environment, University College LondonGower StreetLondonUnited Kingdom

**Keywords:** Ciliates, mating types, mate switching, sex determination, sex ratio, yeast

## Abstract

Predictions about the evolution of sex determination mechanisms have mainly focused on animals and plants, whereas unicellular eukaryotes such as fungi and ciliates have received little attention. Many taxa within the latter groups can stochastically switch their mating type identity during vegetative growth. Here, we investigate the hypothesis that mating type switching overcomes distortions in the distribution of mating types due to drift during asexual growth. Using a computational model, we show that smaller population size, longer vegetative periods and more mating types lead to greater distortions in the distribution of mating types. However, the impact of these parameters on optimal switching rates is not straightforward. We find that longer vegetative periods cause reductions and considerable fluctuations in the switching rate over time. Smaller population size increases the strength of selection for switching but has little impact on the switching rate itself. The number of mating types decreases switching rates when gametes can freely sample each other, but increases switching rates when there is selection for speedy mating. We discuss our results in light of empirical work and propose new experiments that could further our understanding of sexuality in isogamous eukaryotes.

In animals and plants, the extensive diversity of sex determining mechanisms is well known and accompanied by a substantial body of theoretical work to explain their evolution (Bull 1983; Uller and Helanterä 2011; Beukeboom and Perrin 2014; Bachtrog et al. 2014; Van Doorn 2014). This contrasts with the situation in isogamous eukaryotes, such as fungi or ciliates, in which the mechanisms of mating type determination are only characterized for a small subset of taxa, with very few evolutionary hypotheses explaining origins, transitions, and diversity (Iwasa and Sasaki 1987; Perrin 2012; Beukeboom and Perrin 2014; Hadjivasiliou et al. 2015; Hadjivasiliou and Pomiankowski, 2016). One striking mechanism found in members of these groups is mating type switching (Klar 2007; Haber 2012), where mating type identity changes between parents and offspring. Although mating type switching is known from a number of extremely well‐studied organisms such as yeast, its selective advantage is still surprisingly poorly investigated. The current study aims to formally assess when and where mating type switching is selectively advantageous.

Mating type switching, often referred to as stochastic mating type determination, has evolved independently in a number of organisms (Phadke and Zufall 2009; Billiard et al. 2011; Cervantes et al. 2013; Beukeboom and Perrin 2014); for example, in the ciliate, *Tetrahymena thermophila*, mating type is determined stochastically after sex, so that individuals switch between one of seven mating types. Most of the mating type genes exist in a tandem array in the germline “micronucleus.” One of these is activated through recombination, bringing it next to a conserved exon and promoter in the “somatic” macronucleus (Paixão et al. 2011; Cervantes et al. 2013). The same type of stochastic mating type determination appears to be present in some members of the ciliate genus *Paramecium* (Phadke and Zufall 2009). In other taxa within the same genus, mating type is cytoplasmically inherited with high fidelity from the maternal macronucleus (Lepère et al. 2008; Singh et al. 2014), although mating type switching due to environmental influences, such as the circadian rhythm, is known to occur (Barnett 1966; Sawka 2012). Evidence for changes in mating type identity during vegetative growth also exists in the ciliate *Euplotes crassus* (Heckmann 1967). In fungi, mating type switching has been found in the basidiomycete *Agrocybe aegerita* (Labarère and Noël 1992), the filamentous ascomycetes *Chromocrea spinulosa*, *Sclerotinia trgoliorum*, and *Glomerella cingulata* (Perkins 1987), and has evolved in the ascomycete budding yeast *Saccharomyces cerevisiae* (and sister species such as *Hansenula polymorpha* and *Pichia pastoris*; Hanson et al. 2014) and fission yeast *Schizosaccharomyces pombe* (Klar 2007). In these yeasts, individuals contain at least one copy of both mating type alleles, but only express one allele due to a variety of mechanisms such as ectopic recombination and epigenetic silencing (reviewed in Klar 2007; Haber 2012; Hanson et al. 2014). Evidence for some degree of randomization in mating type identity during vegetative growth also exists in the green algae *Chlamydomonas monoica* and *Closterium ehrenbergii*, and the dinoflagellate *Gymnodinium catenatum*, although the switching mechanism in these species is not known (VanWinkle‐Swift and Aubert 1983; Ichimura and Kasai 1995; Figueroa et al. 2010). Finally, we note that despite recent progress (e.g., Beukeboom and Perrin 2014), the sexual lives of many other unicellular eukaryotes remain unknown (Speijer et al. 2015), so more instances of mating type switching are likely to be found in the future.

Why has mating type switching repeatedly evolved? The most prominent hypothesis on the adaptive advantage of mating type switching is the so‐called “lonely spore hypothesis” (Knop 2006; Lin and Heitman 2007; Hanson et al. 2014). This idea grew out of consideration of the life cycle of budding yeasts, in which a diploid parent (heterozygous for mating type alleles α/a) undergoes meiosis and produces an ascus with four haploid spores, half carrying the allele α and half carrying the allele *a*. Typically, the spores germinate and undergo mating with others from the same tetrad, forming two diploid offspring cells. Outcrossing between haploid spores from different asci is possible, but occurs at very low rates in natural populations (Ruderfer et al. 2006; Zeyl and Otto 2007; Tsai et al. 2008). When all spores germinate, the balance of mating types allows all spores to find a sexual partner. However, when one or more spores fail to germinate, some spores are likely to remain unmated. In this situation, mating type switching is advantageous, as it allows unmated haploid cells to divide and change their mating type in a daughter cell, and so quickly generate a compatible partner with whom to pair and restore the diploid condition that is the preferred adult state (Knop 2006; Lin and Heitman 2007).

The “lonely spore hypothesis” is an extreme example of a more general phenomenon, where drift due to small effective population sizes causes distortions in the distribution of mating types (Fisher 1930; Iwasa and Sasaki 1987). Mating type switching could then be selectively advantageous, as it increases the production of cells carrying the rarer mating type, which has higher reproductive value. The genes causing mating type switching will then spread as they become associated with a more even distribution of mating type alleles. However, when mating type switching evolves in response to drift and what rates of switching are selectively favored has yet to be systematically analyzed.

In a recent study, Paixão et al. (2011) modeled the evolution of stochastic mating type determination in *Tetrahymena thermophila*. They showed that in species with multiple mating types, individuals benefit from randomizing the mating type of offspring following sexual reproduction. Such a mechanism counters distortions in the sex ratio due to randomized mating between multiple mating types, and could explain sex determination mechanisms such as those seen in *Tetrahymena thermophila*. Other ciliates and fungi, however, exhibit different mechanisms of mating type switching that involve fewer mating type loci (typically two), in which offspring mating types are not randomly determined, but are assigned the mating type opposite to that of the parental cell (Li et al. 2012). In addition, life cycles of many isogamous species are characterized by multiple clonal cell divisions before sexual reproduction (Ni et al. 2011; Speijer et al. 2015), with drift in the distribution of mating type alleles arising through stochasticity in clonal cell replication. To what extent asexual reproduction modulates the evolution of mating type switching has not been investigated.

In this work, we systematically assess when switching becomes important in countering fluctuations in mating type frequencies. We develop a finite population model to study the evolution of alleles imposing stochastic mating type switching during clonal growth. By explicitly modeling both the asexual and sexual phases of unicellular life cycles, we examine the conditions under which switching is favored. We specifically explore the role of the population size, duration of asexual reproduction and other possible sources of stochasticity that distort the sex ratio. This allows us to make predictions about when switching should evolve and what the expected switching rates would be in real populations.

## The model

To capture the effect of drift on the mating type allele distribution in a finite population with clonal replication, we use individual‐based simulations of a population of *N* haploid cells. Each cell bears two genetically encoded loci. The first locus M codes for an individual's mating type and contains alleles $\left\{M_{1},M_{2},M_{3},...,M_{m}\right\}$ where *m* is a parameter specifying the maximum possible number of different mating types. An individual‐bearing allele $M_{i}$ cannot mate with other individuals bearing the same allele, but is equally likely to mate with others bearing any other mating type allele. A second mating type switching locus S, has a wild‐type allele *S*_1_ and a mutant *S*_2_. These alleles differ in the inheritance of the mating type allele at the locus M (see below). We assume that there is no linkage between the M and S loci.

**Figure 1:**
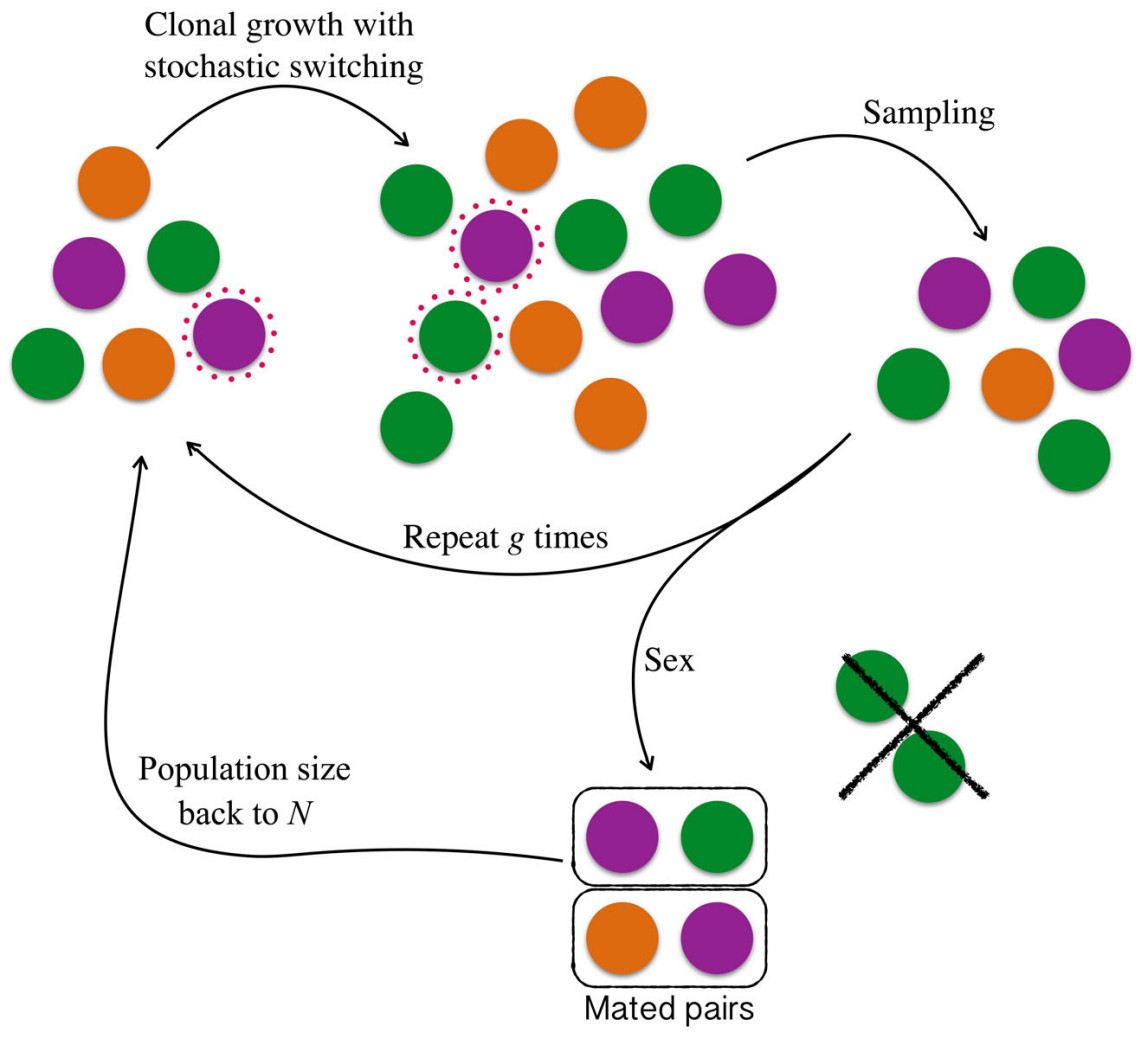
The life cycle considered by the model. Each simulation starts with *N* individuals of *m* mating types at equal proportions indicated by different colors. All individuals grow and divide mitotically to produce two daughters of the same mating type as the parent cell. Individuals that possess the mating type switching gene produce one daughter with a mating type that differs from their own with probability $p_{s}$. In this example, one of the purple cells produces one purple and one green daughter cell indicating a mating type switching event (indicated by the red dotted surround). After growth the population returns to its initial size. This is repeated *g* times. At the end of the vegetative growth cells enter the sexual phase where they form pairs of different mating type until no more heterotypic pairs are possible. Homotypic pairing is not possible. The mated diploids then undergo meiosis back to a haploid state, after which the population size returns to *N* though sampling, and a new vegetative round begins.

A key aspect of the life cycle is that cells undergo *g* rounds of clonal growth before engaging in sexual reproduction (Fig. 1). During a single round of vegetative growth, each cell divides to produce two daughter cells, changing the population size from *N* to 2*N*. We assume that the carrying capacity of the population is fixed and sample without replacement to return the population to *N*. We assume that one daughter cell inherits the parental allele at the M locus. The other daughter cell inherits the parental mating type allele with probability $1-p_{s}$, or a randomly chosen different allele with probability $p_{s}$. The probability $p_{s}$ is determined by the allele at locus S.

Following clonal growth, cells mate with one another at random, subject to the constraint of pairing between cells that are heterotypic at the mating type locus. To give an example of the dynamics during mating, consider a case in which a population contains only two mating type alleles, with *N*_1_ individuals bearing allele *M*_1_ and $N_{2}=N-N_{1}$ individuals bearing allele *M*_2_. If allele *M*_1_ is rarer than *M*_2_ ($N_{1}<N_{2}$), we end up with *N*_1_ randomly chosen pairs of $M_{1}\times M_{2}$ individuals, while the remainder $N_{2}-N_{1}$ individuals bearing the more common allele *M*_2_ are unable to reproduce. After mating, diploid individuals undergo meiosis to give rise to the next generation. If the number of mated pairs is less than N/2, the population is allowed to grow back to carrying capacity (this is implemented in the simulation by sampling with replacement). No switching occurs at the meiotic step. This life cycle encompasses alternating clonal and sexual phases, universal among simple eukaryotes. We also consider several extensions to this simple framework. The life cycle is illustrated schematically in Fig. 1, with model parameters summarized in Table 1.

**Table 1:** Definition of the model parameters

Symbol	Explanation
*N*	Population size
*m*	Number of mating types
*g*	Number of vegetative growth rounds between spells of sexual reproduction
$p_{s}$	The switching rate
S	Switching gene locus
$S_{1},S_{2}$	Wild‐type (no switching) and mutant (switching) genes
*c*	Cost associated with switching
*q*_0_	Initial frequency of the switching gene
*q*_fix_	Probability that the mutant switching allele goes to fixation

Further details about initial conditions and simulation routines are provided in a supplemental text file (Computational Methods). The source code of the individual‐based simulation is written in C++ and can be downloaded here: https://github.com/zenah12/MatingTypeSwitching.

### RESULTS

#### The evolution of mating type switching with two mating types

**Figure 2:**
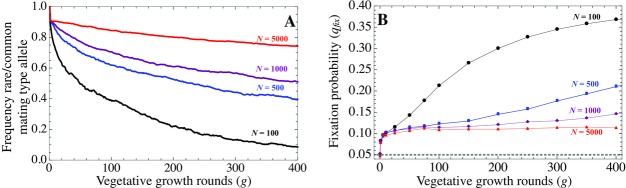
The number of rounds of vegetative growth *g* before each round of sexual reproduction dictates the benefit of switching. (A) In the absence of mating type switching, there is a strong distortion in the distribution of mating types (ratio of the rarest to commonest mating type allele) that increases with *g* (m=2, with equal initial frequencies). (B) The fixation probability of a mutant with switching rate $p_{s}$ = 0.5 introduced in a population in which switching is absent (i.e., with $p_{s}$ = 0). Switching is favored by selection, as fixation rates are higher than expected by drift alone (dotted line). Initial frequency of mutant *q*_0_ = 0.05, as (marked by the gray dotted line). Results are averages over 500 replicate runs in (A) and 50,000 runs in (B).

We first focus on a population with two mating type alleles (m=2). As vegetative growth progresses, asymmetries in the frequency of the two mating type alleles increase (Fig. 2A). Although the distorting effect due to repeated rounds of vegetative growth (*g*) is particularly pronounced when population sizes are small (e.g., N=100), it still leads to considerable distortion in large populations (e.g., *N* = 5000).

To assess the evolution of mating type switching, we measure the fixation probability $q_{fix}$ of a large‐effect mutation *S*_2_ with switching rate $p_{s}$ = 0.5, in a population where switching is absent (i.e., *S*_1_ has $p_{s}$ = 0.0). The mutant *S*_2_ allele is introduced at frequency $q_{0}=0.05$, which determines its fixation probability in the absence of selection (dotted line in Fig. 2B; Wright 1931; Ewens 2010). The difference between $q_{fix}$ and *q*_0_ is indicative of the strength of selection acting on the switching allele. The large‐effect switching mutation is increasingly favored as *g* increases and *N* decreases (Fig. 2B). The probability of fixation of the mating type switching allele is minimally twice that of a neutral allele, even for short vegetative periods and large populations (i.e., *g* =10 and N= 5000 in Fig. 2B).

**Figure 3:**
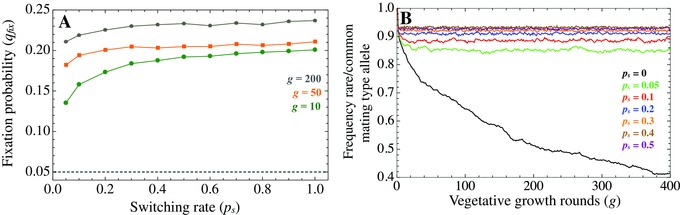
(A) Fixation of mutant switching genes with varying in switching rate $p_{s}$, given different lengths of vegetative growth *g* when compared to the neutral expectation (dotted line). (B) Ratio of the rarest to commonest mating type allele (for m=2) given different values of the switching rate $p_{s}$. Population size *N* = 500. Results are averages over 50,000 replicate runs in (A) and 500 runs in (B).

We next studied the advantage of a range of sequentially increasing values of the switching rates $p_{s}$ in a population in which switching is absent, for three numbers of vegetative growth rounds (*g*= 10, 50, 200), given a fixed population size (*N* = 500) (Fig. 3A). The relative fixation probabilities give a measure of the selective advantage of higher switching rates. For all values of *g*, a mutant with a small switching rate ($p_{s}=0.05$) yields a large fixation probability. Selection acts in favor of further increases in $p_{s}$ but tends to quickly plateau (Fig. 3A). In other words, the switching rate itself becomes irrelevant beyond a value. To understand this, we measured the ratio of the rarest to commonest mating type allele at each vegetative step for a given value of $p_{s}$ (Fig. 3B). This shows that a low switching rate ($p_{s}$ = 0.05) drastically reduces distortions in the distribution of mating type alleles. But further increases in the switching rate confer only a very small additional advantage (Fig. 3B). This suggests that once even minimal levels of switching have evolved, higher switching rates are likely to be only weakly favored.

We also find that varying the number of vegetative growth rounds (*g*) has a minimal effect on selection for higher switching rates (Fig. 3A). Although the fixation probability is higher with more vegetative growth rounds (*g* = 200), the increase in the fixation probability as $p_{s}$ increases is more pronounced with a smaller number of growth rounds (i.e., *g* = 10, Fig. 3A). This seems counterintuitive, as the largest distortions in the frequencies of mating types occur when *g* is large (Fig. 2A). But as selection on switching only acts at sex, longer vegetative growth periods (larger *g*) result in greater drift at the *S* locus. This results in a higher probability of extinction for *S*_2_ mutant alleles as they are initially rare (Fig. S1 and Kimura and Ota 1969). Hence, the combination of a reduced selective advantage for more frequent switching as $p_{s}$ increases, and an increase in drift as *g* becomes larger explains why the fixation probability of higher switching rates levels off more quickly with longer vegetative periods *g* (Fig. 3).

A similar argument can also explain why varying the population size *N* has only minimal effects on the fixation probability of $p_{s}$ mutants (Fig. S2). Although population size has a strong impact on the mating type allele ratio (Fig. 2A), the relative distortion between populations of different sizes decreases once $p_{s}$ > 0.05 (Fig. S2). At the same time, the switching locus *S* undergoes more drift in smaller populations and the probability it goes extinct during vegetative growth increases (Fig. S1; Kimura and Ota 1969). The net effect is a weak impact of *N* on $p_{s}$.

To summarize, our model suggests that relatively low switching rates are sufficient to maintain the mating type ratio near unity in species with two mating types (Fig. 3B), and that switching rates are likely to be lower in populations with prolonged asexual life cycles (Fig. 3A).

#### Restricted evolution of costly mating type switching

Switching is likely to come at a cost. For example, it is well‐known that the presence of switching mechanisms increases DNA replication errors in yeast (Hicks et al. 2010; Gordon et al. 2011). In addition, mating type switching may involve costs associated with replicative delays (Connolly et al. 1988) or costs due to inbreeding in certain contexts (Goddard et al. 2005). To assess the effects of costly mating type switching on the survival probability *f* of an individual cell, we applied a fitness cost that increases with growing rates of switching $p_{s}$,

(1) $$f=1-cp_{s,}^{k}$$

where *c* is a parameter reflecting how strongly the switching rate $p_{s}$ decreases survival, while *k* determines whether costs accelerate (k>1) or decelerate (k<1) with increasing $p_{s}$. Setting k=0 leads to a fixed cost for switching independent of the switching rate. The cost is applied at each asexual growth round. All cells divide to produce a daughter cell leading to a population of size 2*N*, and then are sampled with a probability defined by *f* until the population size returns to *N*.

**Figure 4:**
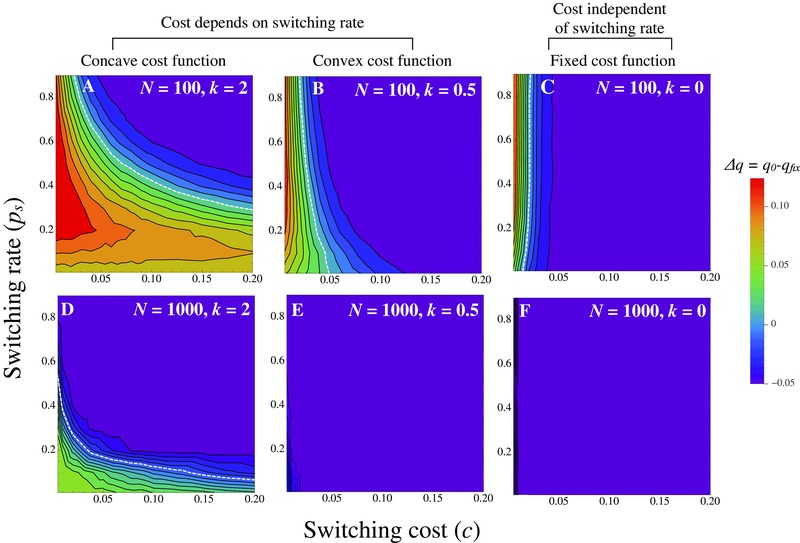
Costs restrict the spread of switching. Heat plots depict the difference in the fixation probability relative to the neutral expectation (defined as $\Delta q=q_{fix}-q_{0}$) for mutants with different switching rates $p_{s}$ and switching costs *c*. We vary the population size (A–C) N=100 and (D–F) N=1000; shape of the cost function (A and D) concave (k=2), (B and E) convex (k=0.5), and (C and F) a fixed cost (k=0). The white dotted lines depict selective neutrality, with mating type switching being selectively favored on the left‐hand size of this line. Parameters: duration of vegetative growth g=50. Results are averaged over 50,000 simulations.

We plot the difference Δq between the initial frequency of the mutant *q*_0_ and its fixation probability *q*_fix_ against (*c*, $p_{s}$) for different population sizes (*N*) and shapes of the cost function (*k*) (Fig. 4). Positive values of Δq (below the white dotted lines in Fig. 4) indicate that switching is selectively favored. When the cost function is concave (costs accelerate with $p_{s}$, k=2) and the population size is small (N=100) we find that higher switching rates ($p_{s}$ > 0.1) are favored, even for high costs of switching (Fig. 4A). The evolution of switching is much more restrictive in larger populations (N=1000) and for decelerating and fixed costs (k=0.5 and k=0, respectively) (Fig. 4B–D). As expected, only small rates of switching are robust to a broad range of costs (e.g., bottom of Fig. 4A). Consequently, when switching imposes significant mutational errors or other types of costs, we only expect populations with a small population size to exhibit high rates of mating type switching.

We also investigated an additional special case where a cost is imposed once per sexual generation. This is equivalent to a cost due to inbreeding after sexual reproduction, following which the resulting cells undergo *g* rounds of asexual cell divisions (Goddard et al. 2005). Now higher costs can be tolerated (since a cost is not being applied continuously during asexual growth), and all values of $p_{s}$ are selectively favored across a range of values for *c* and *k* when N= 100 (Fig. S3).

To conclude, selection on mating type switching becomes more restricted when switching is costly, particularly if costs emerge continuously during the vegetative phase of the life cycle. Costs associated with inbreeding have a less severe impact on the evolution of mating type switching.

#### Continuous evolution of switch rates

**Figure 5:**
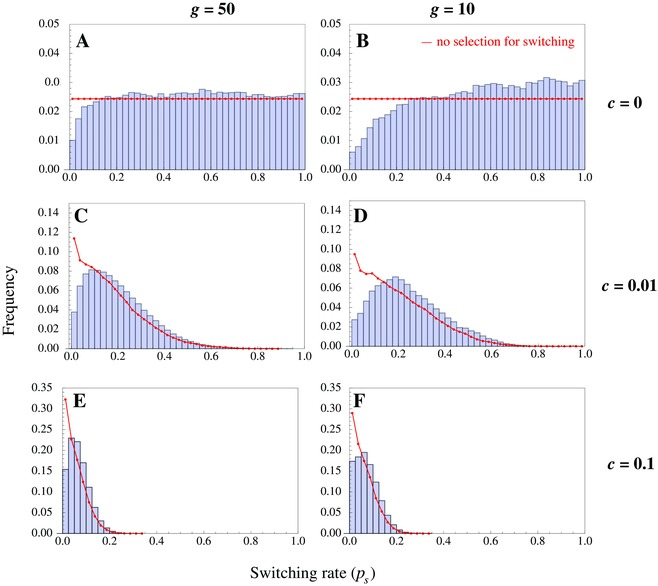
Histograms of the switching rate in a continum‐of‐alleles model for different lengths of vegetative growth (*g*), and switching costs (*c*). Shorter vegetative periods and smaller costs result in more noisy distributions. The red lines indicate the expected frequency distribution for an allele that does not induce mating type switching (but bears the cost of switching). The red lines are exact in panels A and B (uniform distribution expected if no cost) and estimated using simulations in C–F (for a random mutant associated with a small cost defined by *c*). The histograms are plotted by sampling 10^5^ individual instances following the population attaining approximate mutation‐selection balance. Parameters: mutations occur at a rate $\nu =10^{-4}$, with the magnitude of the mutation drawn from a normal distribution with zero mean and standard deviation 0.01. We assumed a concave cost function k=2 and population size *N* = 500.

We show above that incrementally higher rates of switching are only weakly favored (Fig. 3A), suggesting that populations may exhibit substantial variation in $p_{s}$. To study this phenomenon further, we analyze how the distribution of switching rates evolves through time. We allow the switching rate of each cell to mutate at a low rate ν, so that $p_{s}^{\prime }=p_{s}+\varepsilon$, where ε is drawn from a normal distribution with zero mean and standard deviation ξ=0.01, according to a continuum‐of‐alleles model (Kimura and Crow 1964). We then plot $p_{s}$ from a large number of populations subsequent to them reaching mutation‐selection balance (Fig. 5; see SI for simulation details).

When there is no switching cost (*c* = 0), the switching rate is distributed uniformly above a given value or threshold (Fig. 5A–B), corroborating the previous pattern (Fig. 3A). Above the threshold value, changes in $p_{s}$ are effectively neutral. The threshold is lower for more rounds of vegetative growth, as the histograms plateau near $p_{s}=0.2$ for g=50 and $p_{s}=0.5$ for g=10 (Fig. 5A, B). This is due to drift in the value of $p_{s}$ being stronger when the *g* is larger. Both distributions differ from those expected under complete neutrality (red lines; Fig. 5) where any value of $p_{s}$ becomes equally likely.

When a small cost of switching is included (*c* = 0.01), larger values of $p_{s}$ are selected against (Fig. 5C–D). The distribution of $p_{s}$ becomes centered around an optimal value (as determined by mutation, selection, and the switching cost) with fading tails for larger $p_{s}$. Naturally, the distribution of switching rates shrinks and the tails become sharper when the cost rises (Fig. 5E–F). The same is true when *g* increases; longer vegetative periods mean that the ratio of time when switching is costly (during asexual generations) increases relative to the time when switching is selectively advantageous (each sexual round). This pushes $p_{s}$ down explaining the decrease in variance and longer right tails for larger *g* (Fig. 5C, E vs. D, F).

**Figure 6:**
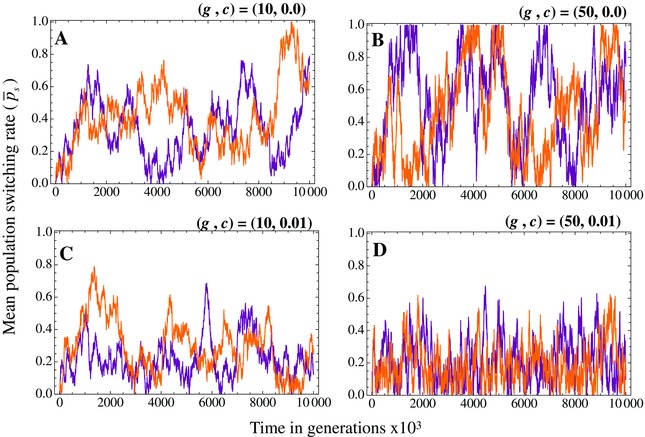
The switching rate for individual populations over time exhibits substantial temporal variation in switching rates, for different vegetative growth periods *g* and switching costs *c*. Each figure shows two randomly sampled replicate runs (different colors). (A) (g,c) = (50, 0), (B) (g,c) = (10, 0), (C) (g,c) = (50, 0.01) and (D) (g,c) = (10, 0.01). Other parameters: mutations occur at a rate $\nu =10^{-4}$, with the magnitude of the mutation drawn from a normal distribution with zero mean and standard deviation 0.01. We assumed a concave cost function k=2 and population size *N* = 500.

Individual simulations show the population average value of the switching rate (${\bar{p}}_{s}$) over time (Fig. 6). The switching rate fluctuates strongly for each of the parameter combinations considered (Fig. 6). Fluctuations are more noticeable in populations that undergo longer asexual phases (Fig. 6A–B). When switching is costly, fluctuations in switching rates are more constrained and frequently hit zero before evolving to nonzero values again (c>0; Fig. 6C–D). In these cases, switching is slightly deleterious during asexual growth and our model predicts that it will be repeatedly lost and regained. Taken together these findings suggest that switching rates may differ substantially between populations, especially if switching comes at a cost and in species that consistently undergo long asexual periods.

#### Multiple mating types

**Figure 7:**
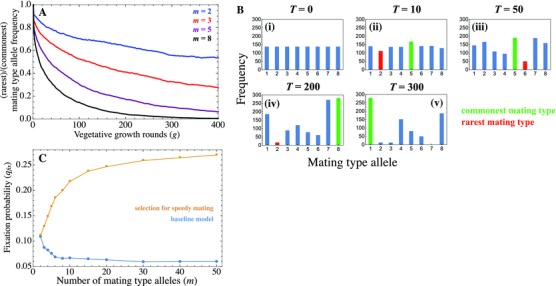
Increasing the number of mating types (*m*) does not favor higher rates of mating type switching, unless other selective forces (such as the speed of mating) play a role. (A) Ratio of the rarest to commonest mating type measured against vegetative growth round (*g*). (B) Distortions in the distribution of mating types in a population with m=8 mating types having undergone *T* rounds of vegetative growth rounds before sex out of a total of 300. No sexual rounds are implemented here. (C) Fixation probability $q_{fix}$ of the switching gene (*S*_2_) against the number of mating types (*m*) using the baseline model (blue) or assuming selection for speedy mating (orange), compared to neutral expectation (dotted line). The switching gene was introduced at $q_{0}=0.05$ with switching rate $p_{s}$ = 0.5. Parameters: N=1000. Results are averaged over 500 simulations in (A) and 50,000 simulations in (C).

Many unicellular eukaryotes have more than two mating types (m>2) (e.g., Billiard et al. 2011; Phadke and Zufall 2009; Beukeboom and Perrin 2014), raising the question how robust our results are to changes in the number of mating types. We varied the number of mating types and show the frequency of the rarest over the most common mating type allele during a single round of clonal growth for populations of size *N* = 1000 (Fig. 7A; equivalent to Fig. 2A with m=2). As the number of mating types increases, the rare‐to‐common allele ratio declines steeply implying that some mating types become very common and others very rare (Fig. 7A–B). This is not surprising, as drift is more potent when there are more mating type alleles for the same number of individuals (Paixão et al. 2011), and eventually drives one or more mating types to extinction (Fig. 7B).

The higher sensitivity of multiple mating types to drift suggests that selection for switching and higher switching rates should be stronger for m>2. Our model predicts exactly the opposite: the fixation probability of the switching allele ($q_{fix}$) declines with increasing number of mating types (blue line in Fig. 7C), implying that selection for mating type switching is, in fact, stronger when m=2. This is corroborated by the finding that the fixation probability plateaus at lower $p_{s}$ for larger *m* (Fig. S4). Why is this so? In the case of two mating types the rarest of the two always enjoys an advantage. But this effect becomes weaker as *m* increases. While asymmetries in frequency between the rarest and commonest mating type increase with higher *m* (cf. Fig. 7A‐B), the commonest mating type can still mate with other, more prevalent mating types, which is not possible when m=2. Consequently, the likelihood of individuals remaining without a compatible partner at the end of mating is lower for higher *m*. The proportion of mated cells increases with *m* (Fig. S5). We thus expect switching to be less favorable as the number of mating types increases, despite the higher likelihood that drift leads to the loss of mating type alleles (Fig. S6).

A previous model has shown that multiple mating types are favored when cells only have time to assess a limited number of potential partners (defined here as speedy mating; Iwasa and Sasaki 1987), as this increases the likelihood that a compatible partner is present within a limited sample of partners. This leads us to ask whether speedy mating increases the evolutionary prospects for mating type switching. We repeated our analysis assuming that gametes that fail to find a compatible partner when first sampled are removed from the gamete pool (Iwasa and Sasaki 1987). This is in contrast to the baseline model that assumes that when two randomly sampled gametes are of the same mating type they are simply returned to the pool of gametes and repeatedly given a chance to mate until no compatible gametes remain. In the case of speedy mating, $q_{fix}$ increases with the number of mating types (Fig. 7C). This is because more common mating types suffer a greater disadvantage when the mating type distribution is more uneven as they are more likely to encounter a cell with the same mating type and so be removed from the mating pool. This effect becomes weaker as the number of mating types increases because the relative frequency of the commonest type decreases with *m*. It leads to a plateau rather than decline in $q_{fix}$ (Fig. 7C) because as the number of mating types increases so does the rate at which mating types are lost during vegetative growth (Fig. S6). Consequently, the presence of multiple mating types and selection for quick partner finding lead to stronger selection for mating type switching. Finally, when m>2 a similar picture emerges for very large populations (*N* = 5000, Fig. S7), suggesting that switching may be adaptive in species with more than two mating types even when population size is large.

## Discussion

In this work, we consider the evolution and consequences of mating type switching in populations with lifestyles reflecting those of many unicellular eukaryotes (Schlegel and Meisterfeld 2003; Ruderfer et al. 2006; Weisse 2008; Doerder 2014). Our work indicates that several aspects of an organism's life history and ecology, such as the population size, duration of asexual reproduction and the number of mating types, contribute to random genetic drift that results in distortion of the mating type allele ratio. Our findings suggest that stochastic mating type switching during asexual growth can help to dampen these distortions and increase the probability of finding compatible partners at sexual reproduction (Figs. 2 and 3). As expected, smaller populations experience more drift and thus have larger distortions in the mating type distribution, leading to stronger selection for switching (Fig. 2B). Also as expected, selection for switching is hindered when switching is costly, particularly when costs increase rapidly and populations are large (Fig. 4). So costly switching is likely to limit the evolution of switching genes and reduce the rate at which switching occurs.

Other factors have more counterintuitive consequences for the evolution of mating type switching. In particular, the length of the vegetative growth period has contrary effects (Figs. 2 and 3). On the one hand, the imbalance in the distribution of mating types increases with the length of the vegetative growth period, as more asexual divisions lead to greater drift (Fig. 2A). On the other hand, selection on the switching gene only acts at sex, as it is only then that individuals with the common mating type compete for partners that have rarer mating types (Fisher 1930; Iwasa and Sasaki 1987). Hence, the length of the vegetative phase dilutes the effect of selection relative to drift. The net effect is that the expected switching rate decreases for longer vegetative phases (Figs. 3A and 5). Moderate switching rates largely mitigate distortions in the distribution of mating type alleles (Fig. 3B), so selection for higher switching rates becomes increasingly weak. This pattern of near neutrality of switching rates above a threshold could lead to temporal variations in the switching rate both within and between populations. Specifically, our model predicts that populations with longer vegetative phases will exhibit larger fluctuations in the switching rate over time (Fig. 6). Switching costs restrict the window over which fluctuations occur and reduce the expected switching rate, but do not abolish the temporal fluctuations associated with longer vegetative phases (Fig. 6).

These results can help in understanding a variety of empirical findings. Studies on fission yeast show that average per‐generation switching rates are typically around ∽0.25 per cell division (Egel 1977; Miyata and Miyata 1981; Klar 2007), while studies on budding yeast report average switching rates close to 0.5 (Hicks and Herskowitz 1976; Nasmyth 1987). Budding yeasts undergo a small number of asexual divisions as haploid spores and mate to return to their diploid state once a potential partner is found (Knop 2006). Therefore, their higher rates of switching are in line with our prediction that higher rates of switching evolve with a lower number of rounds of vegetative growth (Figs. 3 and 5). Fission yeast, on the other hand, proliferate when haploid and only reproduces sexually under stress, and so a lower switching rate suffices to maintain even mating type ratios at sex. Little variation in switching rates has been reported in laboratory strains (patterns of mating type switching appear to be highly replicable (Klar 2007; Haber 2012), although a systematic assessment has yet to be undertaken). Our model predicts that there should be much greater variation in switching rates, with sensitivity to life history and ecological variables. To test the theory developed here, it will be necessary to assess variation in mating type switching among naturally collected strains. This suggests that a variety of species with different life histories need to be examined. The ideas investigated here should also lend themselves to testing using experimental evolution (Kawecki et al. 2012), where switching rate evolution could be monitored in populations forced to undergo vegetative phases of varying lengths. It would be of interest to know whether the mean switching rate evolves in the way suggested by the current model and whether temporal variation indeed typifies the dynamics of switching rate evolution.

The presence of multiple mating types is common among isogamous species (Phadke and Zufall 2009; Billiard et al. 2011; Sawka 2012). We find that drift in the distribution of mating type alleles increases with the number of mating types. Even if all mating types are at equal ratios at the start of each sexual cycle, some are likely to be lost or be rarer than others due to drift during the asexual phase (Fig. 7A–B). But increasing the number of mating types does not necessarily translate into stronger selection for switching. As the number of individuals with each mating type declines with the number of mating types, any distortion making a particular mating type more common causes less of a disadvantage (as it can mate with all others). This effect offsets the greater distortion due to drift with more mating types (Fig. 7C). But this argument supposes that gametes can freely sample each other, and there is no time (or other) penalty in finding a suitable mating partner. If speedy mating is at a premium, as is likely to be the case in many unicellular species (Iwasa and Sasaki 1987), common types have the disadvantage of more frequent encounters with others carrying the same mating type. Under this condition, switching is more strongly favored as individuals from the most common mating type are more likely to encounter one another and so suffer a larger cost in finding a suitable partner. It then follows that selection for switching increases and then plateaus with an increasing number of mating type alleles (Fig. 7C). It would be interesting to further explore the evolution of the number of mating types in populations that sustain switching, but this is outside the interest of the current work.

Our model does not explicitly incorporate a spatial component. For example, some yeasts and other fungi are restricted in their movement and so are likely to find themselves among descendants that share their mating type in the absence of switching. Alternatively when colonies are formed from a single or few individual founders, local mating type distortion is likely to be extreme. Such discrepancies in the mating type ratio are likely to be similar to those caused by the vegetative period in our model. They cause drift in the ratio of different mating types and in the switching locus itself, and so are likely to favor switching. The examination of a spatially explicit extension of our model is needed to fully elucidate how spatial structure influences the evolution of the switching rate.

Some of our findings echo a previous model (Paixão et al. 2011) that considered the sex determination system of *Tetrahymena thermophila*, in which mating type switching (referred to as stochastic mating type determination) occurs once per sexual cycle. This study also reported stronger selection for switching as the number of mating types increased (Paixão et al. 2011). In contrast to our findings, however, Paixão et al. (2011) found that mating type switching is only favored when there are more than two mating types. This is because Paixão et al. (2011) only consider distortions in the mating type allele distribution that result from random mating between multiple mating types (i.e., some mating types mate less often by chance). However, distortions due to random mating necessarily vanish in populations with only two mating types. The novel aspect of the current study is that we identify the asexual growth phase as another, inevitable source of drift that is likely to play a role in many unicellular organisms. This also will impact on species with only two mating types. Furthermore, our model captures the behavior observed in a number of ciliates and yeasts in which mating type switching occurs during the asexual growth period (Labarère and Noël 1992; Klar 2007; Hanson et al. 2014).

Little direct evidence for mating type switching exists in taxa other than fungi or ciliates. Nevertheless, research in some algae and dinoflagellates is suggestive of changes in mating type identity during vegetative growth (VanWinkle‐Swift and Aubert 1983; Ichimura and Kasai 1995; Figueroa et al. 2010). In these studies colonies formed by a single haploid clone exhibit behaviors reminiscent of both mating types and interclonal mating. In addition, sexual fusions appear to be asymmetric (e.g., exhibiting cytoplasmic uniparental inheritance) that led to the hypothesis that individuals carry genes for mating types that are differentially expressed during vegetative growth (VanWinkle‐Swift and Aubert 1983). In *Gymnodinium catenatum*, mating compatibility within a single clone colony is shown to increase over time suggestive of low switching rates (Figueroa et al. 2010). However, the putative switching mechanisms or frequencies at which mating type change takes place are not known in species other than yeasts and some ciliates (Klar 2007; Phadke and Zufall 2009; Sawka 2012). Further studies in algae and dinoflagellates would help determine how general the switching behaviors and mechanisms uncovered in other taxa are, and could contribute significantly in our understanding of sexuality in unicellular species.

In conclusion, our study highlights the importance of drift in the sex ratio and suggests that switching serves to mitigate such distortions, especially in species that undergo occasional sex. We modeled a number of different life history and ecological parameters that can be sources of variation in the mating type ratio. Our work suggests that mating type switching is not just a response to not finding a partner but serves as a mechanism to assure a greater chance of finding a compatible sexual partner. We predict switching events to be more widespread than is currently known. Further work in this direction may help elucidate the relevant selective forces acting on life cycles of isogamous eukaryotes.

## Supporting information


**Figure S1**. Extinction probability of the switching allele *S*_2_ at each step of a single bout of vegetative growth of g=500 rounds, for different initial frequencies *q*_0_.
**Figure S2**. Population size has minimal effects on the fixation probability of switching mutants, once switching rates higher than $p_{s}>0.05$ invade.
**Figure S3**. Inbreeding costs facilitate the spread of switching relative to costs imposed during vegetative growth (cf. Fig. 4).
**Figure S4**. The fixation probability *q*_fix_ rapidly plateaus at lower switch rates $p_{s}$ in populations with a larger number *m* of mating types, relative to those with a smaller number.
**Figure S5**. Increasing the number of mating types *m* increases the proportion of gametes that successfully find a mate.
**Figure S6**. The mean number of mating types present in the population measured against vegetative growth round (*g*), averaged over 500 replicate runs.
**Figure S7**. The fixation probability of the mutant switching allele *S*_2_ plotted against the number of mating types *m* when there is selection for speedy mating.
