# Supplementary material for: The evolution of mating type switching
Source: Evolution. 2016 Jun 17;70(7):1569–81. doi: 10.1111/evo.12959 (PMC5008120; doi:10.1111/evo.12959)
Supplement: Supplementary file 1 — Figure S1. Extinction probability of the switching allele S 2 at each step of a single bout of vegetative growth of g=500 rounds, for different initial frequencies q 0. Figure S2. Population size has minimal effects on the fixation probability of switching mutants, once switching rates higher than ps>0.05 invade. Figure S3. Inbreeding costs facilitate the spread of switching relative to costs imposed during vegetative growth (cf. Fig. 4). Figure S4. The fixation probability q fix rapidly plateaus at lower switch rates ps in populations with a larger number m of mating types, relative to those with a smaller number. Figure S5. Increasing the number of mating types m increases the proportion of gametes that successfully find a mate. Figure S6. The mean number of mating types present in the population measured against vegetative growth round (g), averaged over 500 replicate runs. Figure S7. The fixation probability of the mutant switching allele S 2 plotted against the number of mating types m when there is selection for speedy mating. [file EVO-70-1569-s001.zip › evo12959-sup-0001-SupMat.pdf]

Computational Methods: The Evolution of Mating Type Switching

Zena Hadjivasiliou[1,2*], Andrew Pomiankowski[1,2*] and Bram Kuijper[1,2]

1 Computational methods

1.1 Life cycle

We model N haploid cells so that each cell is individually characterized by a mating type locus $\mathcal{M}$ (alleles of which can take values $\{M_1, M_2, M_3, \ldots, M_m\}$ where m is the total number of mating types), and a switching locus $\mathcal{S}$, with alleles that can take values in $[0, 1]$, reflecting the switching probability p_s. Our simulation consists of an $N \times 2$ matrix that stores the mating type and switching alleles for each individual cell.

At each vegetative growth round each cell divides and the population size changes from N to $2N$. Cells whose value at $\mathcal{S}$ is equal to $p_s = 0$ produce two daughter cells who both inherit their parental mating type. Cells with $p_s > 0$ produce one daughter cell with the same mating type as themselves, while the other daughter cell inherits the parental mating type with probability $1 - p_s$ or obtain a randomly chosen mating type with probability p_s . The allele at the switching locus S is inherited clonally by both daughter cells. We then sample without replacement to return to a population os size N.

Sexual reproduction occurs by randomly sampling two individuals from the pool of N unmated individuals, and allowing mating if they have different mating type alleles. Mated pairs are removed from the population of unmated individuals. The mating process is then repeated until all remaining individuals in the unmated pool have the same mating type. Each mated pair then produces two haploid daughter cells. We assume full recombination between the $\mathcal{M}$ and $\mathcal{S}$ loci. No mating type switching is allowed at this stage. If

some individuals are unmated, the population size will consist of less than N individuals after sex. To return the population to N, we sample with replacement until the population size returns to carrying capacity.

1.2 Mutation and estimation of q_{fix}

At the start of each simulation, all mating type alleles M_i have the same frequency of N/m for all i, while the switching locus $\mathcal{S}$ initially only bears the wild type allele S_1 coding for no switching ($p_s = 0$). The mutant allele S_2 is introduced in each cell with with probability q_0 during the first vegetative phase of the life cycle, at step $g-t$ (we set $t = 5$ when $g < 5$, and $t = 0$ otherwise). This treatment is used to ensure that the mutant is introduced late in the first life cycle and to avoid large discrepancies due to varying population size N and duration of the vegetative growth phase g early on in the analysis. To calculate the fixation probability q_{fix} of the mutant switching allele S_2 , the process of vegetative growth followed by sexual reproduction is iterated until the frequency of the mutant allele reaches fixation or is lost. Each simulation is replicated a large number of times (see the legends of individual figures for the number of replications used). q_{fix} is estimated from the number of fixation events divided by the number of independent simulations.

1.3 Computing the ratios of commonest to rarest alleles

To compute the rarest to commonest mating type alleles plots we allow a population where initially all mating type alleles M_i have the same frequency of N/m for all i to undergo g vegetative steps, where the sampling method described in 1.1 is applied. The frequency of each mating type following each vegetative step is recorded so that each independent replicate run results in an M_i x g matrix with the frequencies of mating types over time. We compute the frequency of the rarest over the commonest mating type allele at each time step and then average over all replicate runs (the number of repeats is given in the figure legends). The plots in Fig. 3B and Fig. S2 assume that switching occurs during vegetative growth as indicated by the value of p_s.

1.4 Costly switching

We implemented costly switching by introducing a bias during vegetative growth. The population doubles as before but the sampling process to return to population of size N now depends on the switching locus. Cells are sampled without replacement with a probability f where,

$$f = 1 - c p_s^k$$

where c is a parameter reflecting how strongly the switch rate p_s decreases survival, while k determines whether costs accelerate ($k > 1$) or decelerate ($k < 1$) with increasing p_s. Setting $k = 0$ leads to a fixed cost for cells carrying the switching genes independently of the switching rate. This cost is implemented at each vegetative step for the panels in Fig. 4, and only once after each sexual round in Fig. S3.

1.5 Continuum of alleles method

We used a continuum-of-alleles model to assess the continuous evolution of switch rates p_s (Fig. 5 and Fig. 6). We allow the switching rate of each cell to mutate at each vegetative time step prior to the population doubling at a low rate $\nu = 10^{-4}$, so that $p_s' = p_s + \epsilon$, where ϵ is drawn from a normal distribution with zero mean and standard deviation $\xi = 0.01$. When mutation results in $p_s < 0$ we set $p_s = 0$, and similarly when the mutated $p_s > 1$ we set $p_s = 1$. We then plot histograms of the value of p_s (averaged over 10^5 replicates) at a time point subsequent to them reaching mutation-selection balance. We assume that mutation-selection balance is achieved when the mean value p_s averaged over all 10^5 replicates stabilizes. This was achieved after a maximum of 800,000 generations so we used this as a global threshold for all 10^5 replicates used to plot the histograms. The red lines in histograms A and B indicate the expected distribution of a neutral trait taking values in [0, 1]: for a neutral trait we expect to see a uniform distribution in a continuum-of-alleles model. The red lines in C-F show expected distribution for a trait taking values in [0, 1] which is slightly deleterious as defined by our cost function, but that confers no switching. All histograms and the red line estimates were computed at the mutation-selection balance as defined above, using 10^5 independent repeats.
