# Supplementary figures and images for: The evolution of mating type switching
Source: Evolution. 2016 Jun 17;70(7):1569–81. doi: 10.1111/evo.12959 (PMC5008120; doi:10.1111/evo.12959)

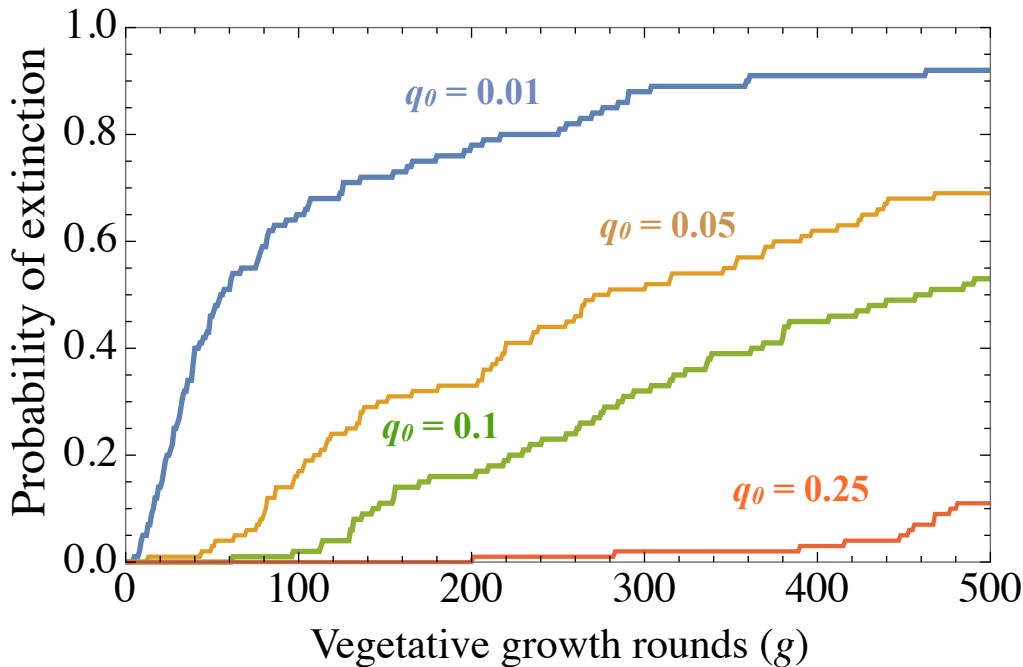

Supplement: Supplementary file 1 — Figure S1. Extinction probability of the switching allele S 2 at each step of a single bout of vegetative growth of g=500 rounds, for different initial frequencies q 0. Figure S2. Population size has minimal effects on the fixation probability of switching mutants, once switching rates higher than ps>0.05 invade. Figure S3. Inbreeding costs facilitate the spread of switching relative to costs imposed during vegetative growth (cf. Fig. 4). Figure S4. The fixation probability q fix rapidly plateaus at lower switch rates ps in populations with a larger number m of mating types, relative to those with a smaller number. Figure S5. Increasing the number of mating types m increases the proportion of gametes that successfully find a mate. Figure S6. The mean number of mating types present in the population measured against vegetative growth round (g), averaged over 500 replicate runs. Figure S7. The fixation probability of the mutant switching allele S 2 plotted against the number of mating types m when there is selection for speedy mating. [file EVO-70-1569-s001.zip › evo12959-sup-0002-FigS1.pdf]

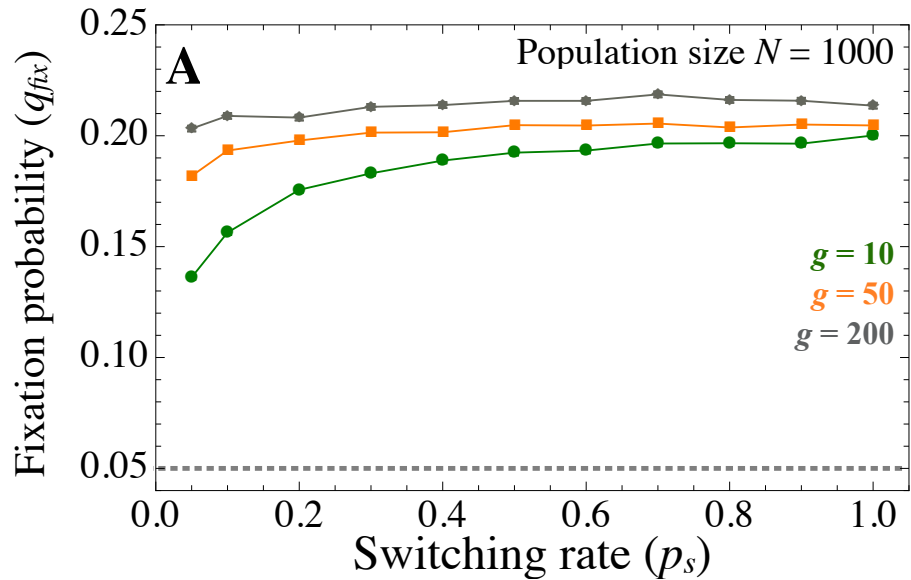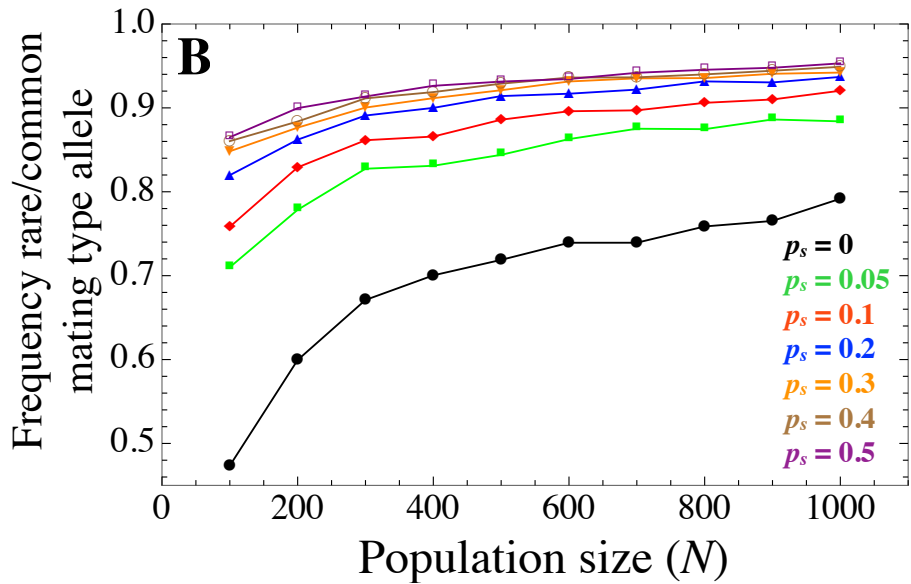

Supplement: Supplementary file 1 — Figure S1. Extinction probability of the switching allele S 2 at each step of a single bout of vegetative growth of g=500 rounds, for different initial frequencies q 0. Figure S2. Population size has minimal effects on the fixation probability of switching mutants, once switching rates higher than ps>0.05 invade. Figure S3. Inbreeding costs facilitate the spread of switching relative to costs imposed during vegetative growth (cf. Fig. 4). Figure S4. The fixation probability q fix rapidly plateaus at lower switch rates ps in populations with a larger number m of mating types, relative to those with a smaller number. Figure S5. Increasing the number of mating types m increases the proportion of gametes that successfully find a mate. Figure S6. The mean number of mating types present in the population measured against vegetative growth round (g), averaged over 500 replicate runs. Figure S7. The fixation probability of the mutant switching allele S 2 plotted against the number of mating types m when there is selection for speedy mating. [file EVO-70-1569-s001.zip › evo12959-sup-0003-FigS2.pdf]

Inbreeding cost

Concave cost function

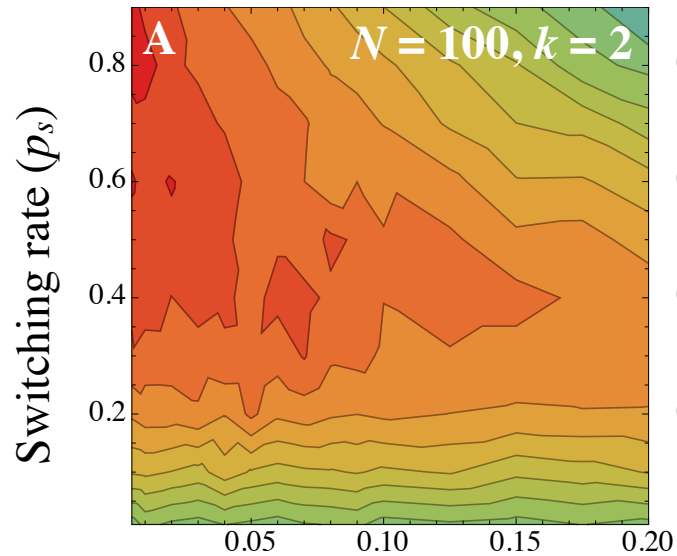

Convex cost function

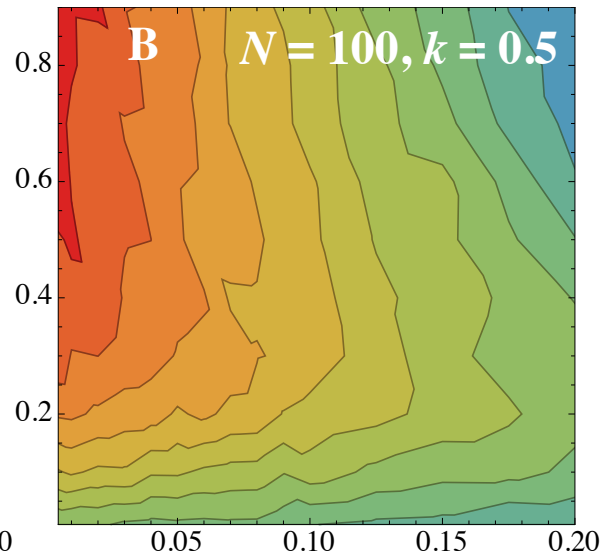

Fixed cost

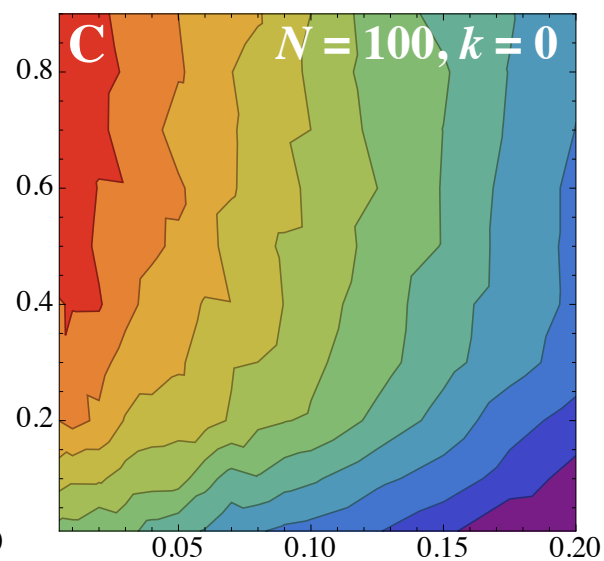

$\Delta q = q_0 - q_{fix}$

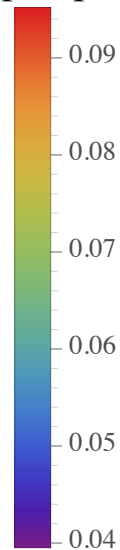

Supplement: Supplementary file 1 — Figure S1. Extinction probability of the switching allele S 2 at each step of a single bout of vegetative growth of g=500 rounds, for different initial frequencies q 0. Figure S2. Population size has minimal effects on the fixation probability of switching mutants, once switching rates higher than ps>0.05 invade. Figure S3. Inbreeding costs facilitate the spread of switching relative to costs imposed during vegetative growth (cf. Fig. 4). Figure S4. The fixation probability q fix rapidly plateaus at lower switch rates ps in populations with a larger number m of mating types, relative to those with a smaller number. Figure S5. Increasing the number of mating types m increases the proportion of gametes that successfully find a mate. Figure S6. The mean number of mating types present in the population measured against vegetative growth round (g), averaged over 500 replicate runs. Figure S7. The fixation probability of the mutant switching allele S 2 plotted against the number of mating types m when there is selection for speedy mating. [file EVO-70-1569-s001.zip › evo12959-sup-0004-FigS3.pdf]

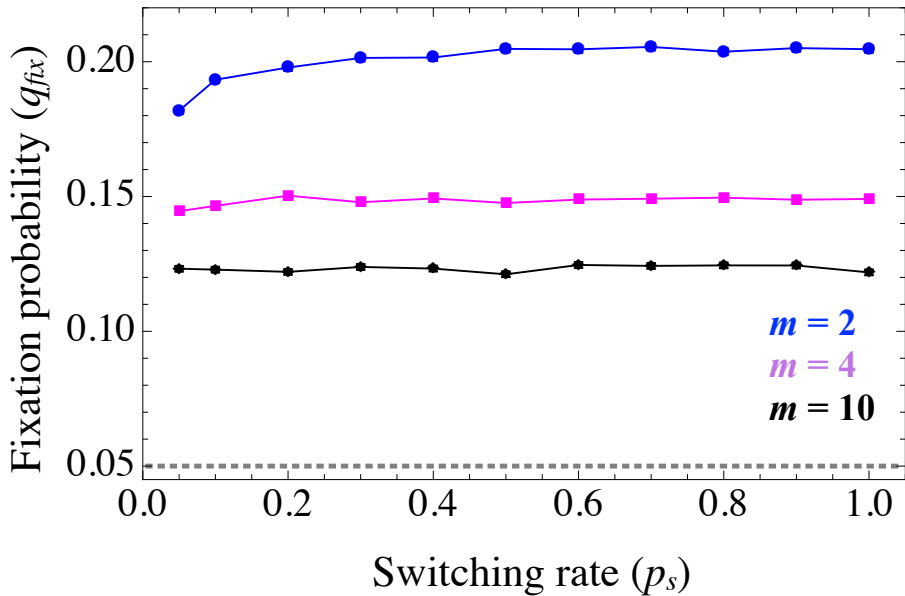

Supplement: Supplementary file 1 — Figure S1. Extinction probability of the switching allele S 2 at each step of a single bout of vegetative growth of g=500 rounds, for different initial frequencies q 0. Figure S2. Population size has minimal effects on the fixation probability of switching mutants, once switching rates higher than ps>0.05 invade. Figure S3. Inbreeding costs facilitate the spread of switching relative to costs imposed during vegetative growth (cf. Fig. 4). Figure S4. The fixation probability q fix rapidly plateaus at lower switch rates ps in populations with a larger number m of mating types, relative to those with a smaller number. Figure S5. Increasing the number of mating types m increases the proportion of gametes that successfully find a mate. Figure S6. The mean number of mating types present in the population measured against vegetative growth round (g), averaged over 500 replicate runs. Figure S7. The fixation probability of the mutant switching allele S 2 plotted against the number of mating types m when there is selection for speedy mating. [file EVO-70-1569-s001.zip › evo12959-sup-0005-FigS4.pdf]

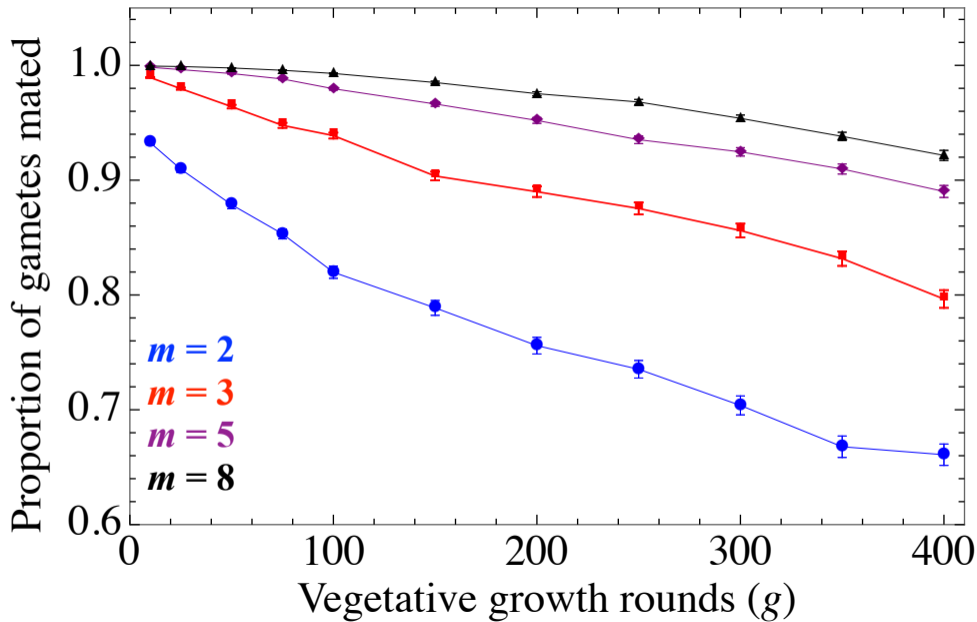

Supplement: Supplementary file 1 — Figure S1. Extinction probability of the switching allele S 2 at each step of a single bout of vegetative growth of g=500 rounds, for different initial frequencies q 0. Figure S2. Population size has minimal effects on the fixation probability of switching mutants, once switching rates higher than ps>0.05 invade. Figure S3. Inbreeding costs facilitate the spread of switching relative to costs imposed during vegetative growth (cf. Fig. 4). Figure S4. The fixation probability q fix rapidly plateaus at lower switch rates ps in populations with a larger number m of mating types, relative to those with a smaller number. Figure S5. Increasing the number of mating types m increases the proportion of gametes that successfully find a mate. Figure S6. The mean number of mating types present in the population measured against vegetative growth round (g), averaged over 500 replicate runs. Figure S7. The fixation probability of the mutant switching allele S 2 plotted against the number of mating types m when there is selection for speedy mating. [file EVO-70-1569-s001.zip › evo12959-sup-0006-FigS5.pdf]

Mean number of mating  
types in population

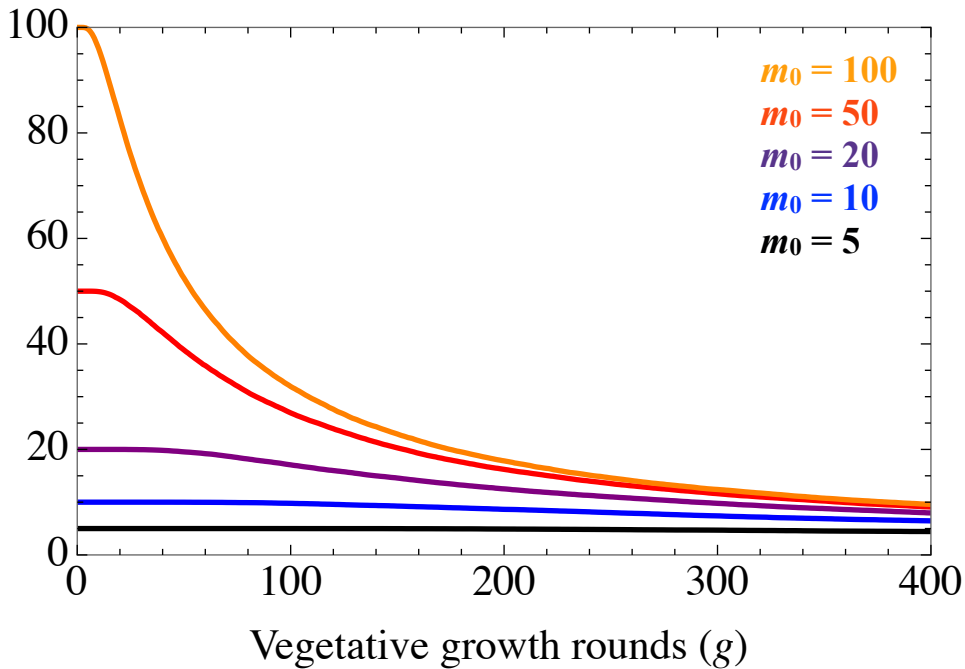

Supplement: Supplementary file 1 — Figure S1. Extinction probability of the switching allele S 2 at each step of a single bout of vegetative growth of g=500 rounds, for different initial frequencies q 0. Figure S2. Population size has minimal effects on the fixation probability of switching mutants, once switching rates higher than ps>0.05 invade. Figure S3. Inbreeding costs facilitate the spread of switching relative to costs imposed during vegetative growth (cf. Fig. 4). Figure S4. The fixation probability q fix rapidly plateaus at lower switch rates ps in populations with a larger number m of mating types, relative to those with a smaller number. Figure S5. Increasing the number of mating types m increases the proportion of gametes that successfully find a mate. Figure S6. The mean number of mating types present in the population measured against vegetative growth round (g), averaged over 500 replicate runs. Figure S7. The fixation probability of the mutant switching allele S 2 plotted against the number of mating types m when there is selection for speedy mating. [file EVO-70-1569-s001.zip › evo12959-sup-0007-FigS6.pdf]

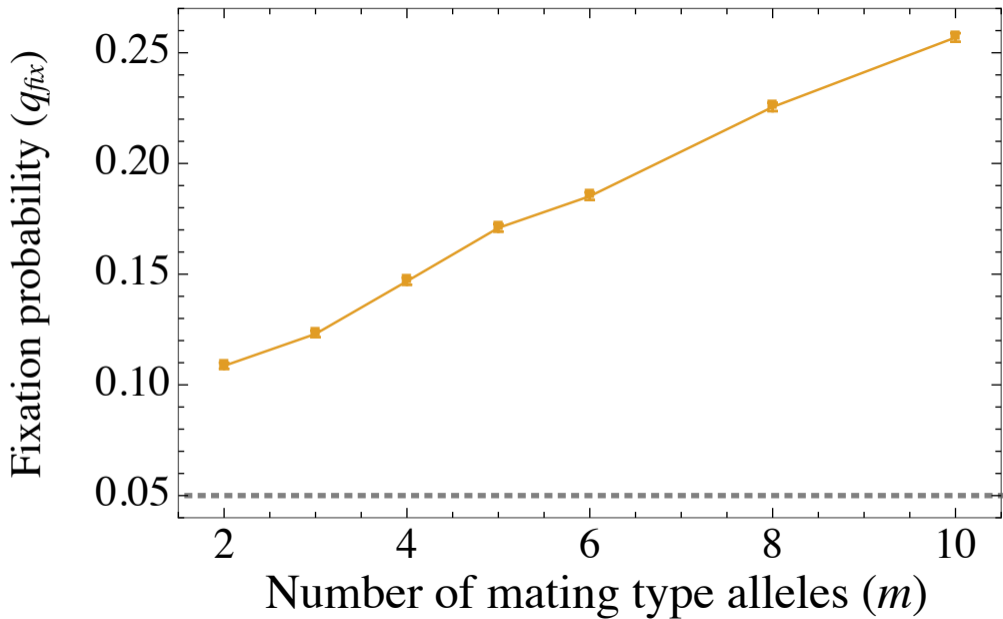

Supplement: Supplementary file 1 — Figure S1. Extinction probability of the switching allele S 2 at each step of a single bout of vegetative growth of g=500 rounds, for different initial frequencies q 0. Figure S2. Population size has minimal effects on the fixation probability of switching mutants, once switching rates higher than ps>0.05 invade. Figure S3. Inbreeding costs facilitate the spread of switching relative to costs imposed during vegetative growth (cf. Fig. 4). Figure S4. The fixation probability q fix rapidly plateaus at lower switch rates ps in populations with a larger number m of mating types, relative to those with a smaller number. Figure S5. Increasing the number of mating types m increases the proportion of gametes that successfully find a mate. Figure S6. The mean number of mating types present in the population measured against vegetative growth round (g), averaged over 500 replicate runs. Figure S7. The fixation probability of the mutant switching allele S 2 plotted against the number of mating types m when there is selection for speedy mating. [file EVO-70-1569-s001.zip › evo12959-sup-0008-FigS7.pdf]
